# Effects of the brown algae *Sargassum horneri* and *Saccharina japonica* on survival, growth and resistance of small sea urchins *Strongylocentrotus intermedius*

**DOI:** 10.1038/s41598-020-69435-8

**Published:** 2020-07-27

**Authors:** Fangyuan Hu, Mingfang Yang, Peng Ding, Xu Zhang, Zhouling Chen, Jingyun Ding, Xiaomei Chi, Jia Luo, Chong Zhao, Yaqing Chang

**Affiliations:** Key Laboratory of Mariculture & Stock Enhancement in North China’s Sea, Ministry of Agriculture, Dalian Ocean University, Dalian, 116023 China

**Keywords:** Fisheries, Behavioural ecology, Marine biology

## Abstract

Mass mortality of the long line culture of the sea urchin *Strongylocentrotus intermedius* in summer, which is greatly associated with their disease, energy storage and resistant abilities, is the most serious problem for the development of the aquaculture. Here, a feeding experiment was conducted for ~ 9 weeks to investigate the survival, growth and gonadal development of small *S*. *intermedius* (~ 3 cm) fed either brown algae *Sargassum horneri* or *Saccharina japonica*. Subsequently, we assessed their resistant abilities via observing the behaviors of righting, tube feet extension and Aristotle's lantern reflex at both moderately elevated and acutely changed water temperatures. Sea urchins fed *S*. *horneri* showed significantly fewer diseased individuals and slower gonadal development than those fed *S*. *japonica*. Consistently, significantly greater Aristotle's lantern reflex occurred in sea urchins fed *S*. *horneri* at moderately elevated temperatures. These findings suggest that *S*. *horneri* has direct application potential as food for the long line culture of *S*. *intermedius* in summer because of the advantage in health, energy storage (avoid the energy loss caused by gonadal development at small body sizes) and resistance abilities. In comparison, sea urchins fed *S*. *japonica* outperformed those fed *S*. *horneri* for all experimental behaviors under the acutely changed water temperatures. These findings clearly suggest that *S*. *intermedius* fed *S*. *japonica* is more suitable for the areas with cold water mass in summer, because it can effectively avoid or reduce the negative impacts of acute changes of water temperature on sea urchins. The present study provides valuable information into the management of the long line culture of *S*. *intermedius* in summer.

## Introduction

The sea urchin *Strongylocentrotus intermedius* is a seafood delicacy with high commercial value in the world^1,2^. Interest in *S. intermedius* aquaculture has grown rapidly in the last decade^3,4^. The annual production of sea urchins, for example, was over 8,844 tons in China in 2018^5^. Long line culture is the most important approach to meeting market demands for *S. intermedius*^1^. The brown alga *Saccharina japonica* is commonly used as food for *S*. *intermedius* aquaculture in Japan^3^ and northern China^6^. However, fresh *S*. *japonica* is largely unavailable in summer and thus dried *S*. *japonica* is used for food at that season^1^. Our previous study found that the brown alga *Sargassum horneri* is an alternative diet for *S*. *japonica* and consequently addressed the food shortage problem for the aquaculture of *S*. *intermedius* in summer^7^. However, mass mortality of the long line culture of *S. intermedius* in summer, which is greatly associated with their disease, energy storage, resistant abilities^1^, is the most serious unaddressed problem for the aquaculture of *S. intermedius*^8^.

Reproduction greatly consumes stored energy and is probably associated with various diseases even death in commercially important marine invertebrates such as the oyster *Crassostrea gigas*^9^. Cultured *S*. *intermedius* (~ 3 cm of test diameter) showed precocious gonads in July in northern China^10^, suggesting that the excessive allocation of energy to gametogenesis may be responsible for the mortality of sea urchins in summer. *Strongylocentrotus intermedius* fed *S*. *horneri* showed significantly lower gonad yield than those fed *S*. *japonica* in summer^7^. Low gonad production commonly results in less gametogenesis in sea urchin gonads^1,11^. Therefore, *S*. *horneri* is probably valuable to prevent precocious puberty of *S*. *intermedius* and subsequently to enhance the energy storage.

In addition, long line cultured *S*. *intermedius* are frequently exposed to local unfavorable water temperatures^12^. It has been well documented that *S*. *intermedius* are greatly impacted by elevated water temperatures in summer^3,13^. Besides, acute changes of water temperature also result in negative impacts on regional aquaculture^14,15^. For example, water temperature frequently decreases rapidly from 22 to 16 °C by the cold water mass at Haiyang island near Dalian (39° 03′ N, 123°09′ E) in summer^16^, causing mass mortality of cultured species such as the scallop *Chlamys farreri*^17^. Enhancing resistance abilities can improve the survival of sea urchins at adverse water temperatures. Food is one of the most important methods to facilitate biological resistance abilities^18,19^. Dietary intervention successfully reduced the mortality of aquacultural Australian greenlip abalone *Haliotis laevigata* in unfavorable conditions^20^. However, whether *S*. *horneri* and *S*. *japonica* have potential application for improving the resistance abilities of *S*. *intermedius* to adverse water temperatures is not known.

Sea urchins have fitness-related behaviors including righting, tube feet extension and Aristotle's lantern reflex. Righting behavior refers to an inverted sea urchin placed on its aboral surface to correct the posture with the aboral side up^21,22^ and has been commonly using as a stress indicator^23–25^. Tube feet extension, which refers to the ability of sea urchins to extend their tube feet^26^, is important for the fitness of sea urchins^27^. The Aristotle's lantern reflex is the process of opening and closing of the teeth, which affects the capacity of sea urchins to grasp food with their teeth^28,29^.

The main purposes of the present study are to investigate: (1) whether *S*. *horneri* decreases the mortality and morbidity of sea urchins, compared with *S*. *japonica*; (2) whether *S*. *horneri* slows the gonadal development of *S*. *intermedius*, compared with *S*. *japonica*; (3) whether *S*. *horneri* and *S*. *japonica* have potential application for improving the resistance of *S*. *intermedius* under adverse temperatures.

## Results

### Crude protein, fiber, fat and ash of *S*. *horneri* and *S*. *japonica*

The concentrations of crude protein (186.33 ± 2.66 g/kg) and crude fat (10.00 ± 4.36 g/kg) of dried *S*. *horneri* were not significantly different from those of dried *S*. *japonica* (210.50 ± 6.15 g/kg, *t* = 1.240, *P* = 0.283 for crude protein; 6.00 ± 2.83 g/kg, *t* = 1.315, *P* = 0.259 for crude fat). However, the concentrations of crude fiber (46.67 ± 6.80 g/kg) and ash (13.94 ± 4.02 g/kg) of dried *S*. *horneri* were significantly larger than those of dried *S*. *japonica* (31.50 ± 9.19 g/kg, *t* = 2.910, *P* = 0.044 for crude fiber; 1.95 ± 0.39 g/kg, *t* = 5.128, *P* = 0.007 for ash).

### Experiment I

#### Number of survivors and diseased sea urchins

Diets did not significantly affect the number of survivors (*χ*^2^ = 0.116, *P* = 1.000, Table 1). However, *S*. *intermedius* fed *S*. *horneri* showed significantly fewer diseased individuals than those fed *S*. *japonica* (*χ*^2^ = 4.421, *P* = 0.036, Table 1).

**Table 1:** Differences in the number of survivors and diseased sea urchins between the treatments in summer.

	*Sargassum horneri*	*Saccharina japonica*	*χ*^2^	*P*
Survivors	96	95	0.116	1.000
Dead	4	5		
Diseased	8	18	4.421	0.036
Not diseased	92	82		

*Sargassum horneri* and *Saccharina japonica* refer to the experimental and control groups, respectively. *χ*^2^ and *P* were the statistical results of the Fisher′s exact test. A probability level of *P* < 0.05 was considered significant.

#### Dried food consumption

**Figure 1:**
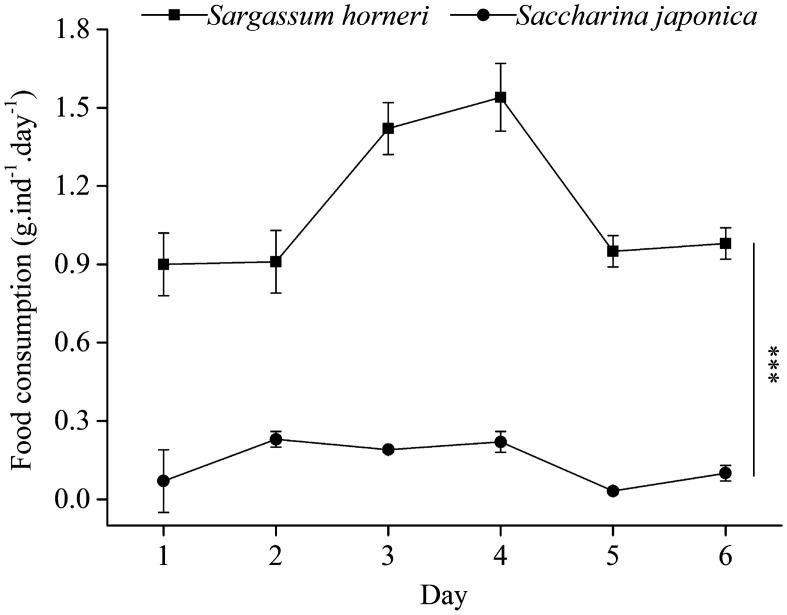
Dried food consumption (g ind^−1^ day^−1^) of *Strongylocentrotus intermedius* fed different diets for six consecutive days (mean ± SD, N = 5). *Sargassum horneri* and *Saccharina japonica* refer to the experimental and control groups, respectively. The asterisks *** mean *P* < 0.001.

The average dried food consumption of *S*. *intermedius* fed *S*. *horneri* (1.12 ± 0.29 g ind^−1^ day^−1^) was significantly more than those fed *S*. *japonica* (0.14 ± 0.08 g ind^−1^ day^−1^, *P* < 0.001, Fig. 1).

#### Test diameter and wet body weight

Compared with the initial conditions, test diameter significantly increased in both treatments (*t* = 4.272, *P* < 0.001 for *S*. *intermedius* fed *S*. *horneri*; *t* = 5.791, *P* < 0.001 for individuals fed *S*. *japonica*). However, there was no pronounced difference in wet body weight between groups (*t* = 1.784, *P* = 0.081 for *S*. *intermedius* fed *S*. *horneri*; *t* = 1.002, *P* = 0.321 for individuals fed *S*. *japonica*).

After ~ 9 weeks, test diameter and wet body weight of *S*. *intermedius* fed *S*. *horneri* (30.09 ± 2.93 mm for test diameter; 9.86 ± 2.34 g for wet body weight) were not significantly different from those fed *S*. *japonica* (30.19 ± 1.87 mm, *t* = 0.162, *P* = 0.872 for test diameter; 9.21 ± 1.48 g, *t* = 1.237, *P* = 0.223 for wet body weight).

#### Specific growth rate

There was no significant difference in specific growth rate (0.188 ± 0.189 for *S*. *intermedius* fed *S*. *horneri*; 0.080 ± 0.192 for individuals fed *S*. *japonica*) between both diet treatments (*t* = 0.891, *P* = 0.399).

#### Wet gut weight

No significant difference in wet gut weight was detected between treatments (0.19 ± 0.05 g for *S*. *intermedius* fed *S*. *horneri*; 0.13 ± 0.05 g for sea urchins fed *S*. *japonica*; *t* = 1.900, *P* = 0.087).

#### Aristotle's lantern length and wet Aristotle's lantern weight

Aristotle's lantern length (7.88 ± 0.74 mm) and wet Aristotle's lantern weight (0.46 ± 0.05 g) of *S*. *intermedius* fed *S*. *horneri* were not significantly different from those fed *S*. *japonica* (7.97 ± 0.34 mm, *t* = 0.275, *P* = 0.789 for Aristotle's lantern length; 0.40 ± 0.06 g, *t* = 2.036, *P* = 0.069 for wet Aristotle's lantern weight).

#### Gonadal yield

Compared with the initial conditions, no significant differences were found in wet gonad weight (*t* = 0.197, *P* = 0.846 for *S*. *intermedius* fed *S*. *horneri*; *t* = 0.491, *P* = 0.631 for individuals fed *S*. *japonica*) and gonad index (*t* = 0.281, *P* = 0.783 for *S*. *intermedius* fed *S*. *horneri*; *t* = 0.435, *P* = 0.670 for individuals fed *S*. *japonica*) in either treatment.

Significant differences were not detected in wet gonad weight (0.58 ± 0.45 g for *S*. *intermedius* fed *S*. *horneri*; 0.63 ± 0.35 g for individuals fed *S*. *japonica*; *t* = 0.195, *P* = 0.850) and gonad index (5.83 ± 1.90 for *S*. *intermedius* fed *S*. *horneri*; 7.17 ± 1.40 for individuals fed *S*. *japonica*; *t* = 0.564, *P* = 0.585) in either treatment.

#### Gonadal development

After ~ 9 weeks, all of the gonads of *S*. *intermedius* fed *S*. *horneri* were in the growing stage (stage II). However, 83.33% and 16.67% of individuals fed *S*. *japonica* were in the premature gonads (stage III) and growing stage (stage II), respectively.

**Figure 2:**
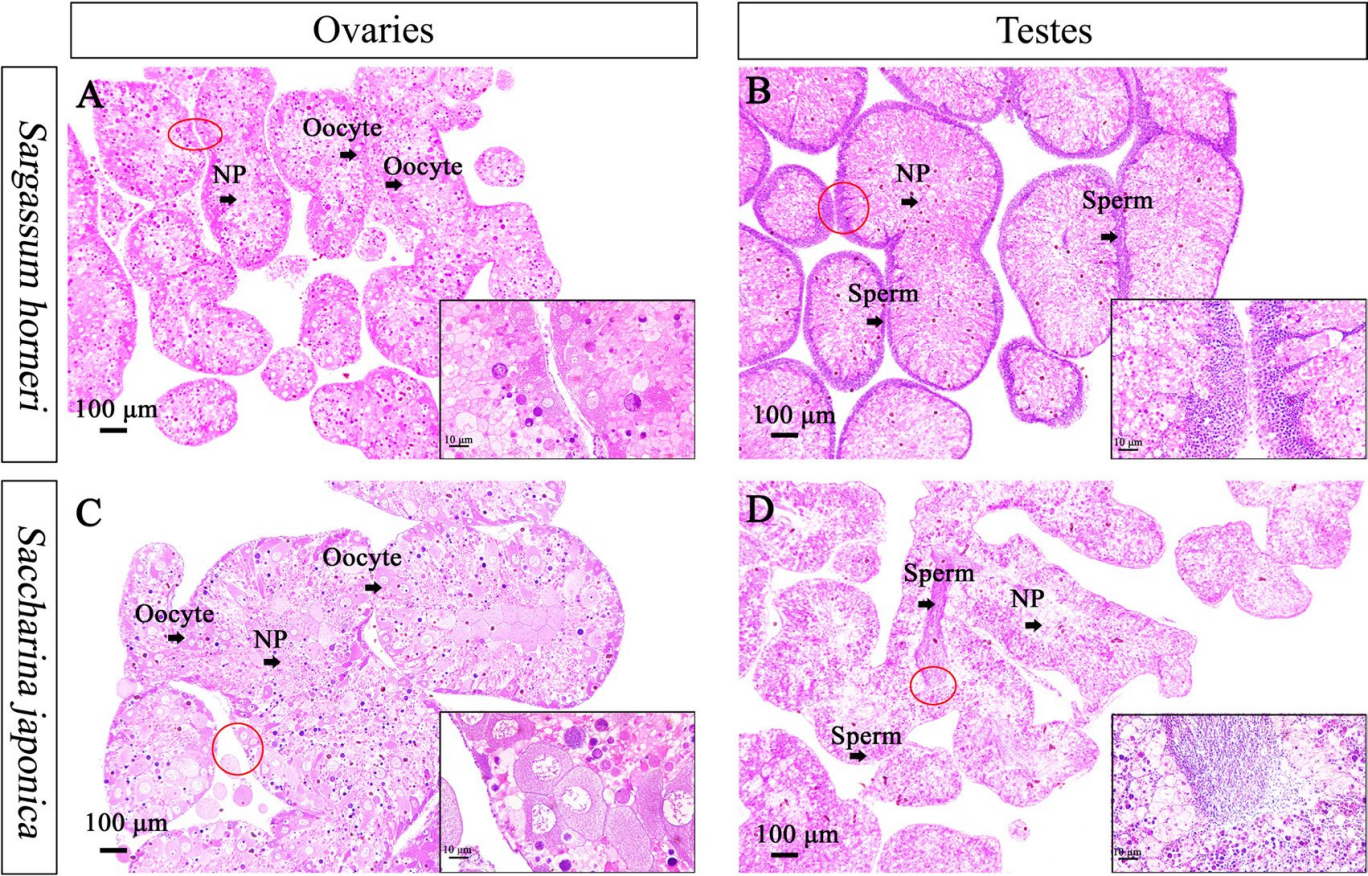
Histology of ovaries (**A**,**C**) and testes (**B**,**D**) of *Strongylocentrotus intermedius* (N = 6) fed different brown algae after ~ 9 weeks. *Sargassum horneri* and *Saccharina japonica* refer to the experimental and control groups, respectively. In the gonads of sea urchins in the experimental group, the primary oocytes only attached to the follicular wall of ovaries (**A**) and sperms only occurred in the follicular wall of testes (**B**). Regarding the gonads of sea urchins in the control group over the same period, the oocytes were detached from the wall and gradually replaced nutritive phagocytes in the follicular cavity (**C**). Consistently, the basophilic clusters of sperm with a length of ~ 2 μm occurred in both the follicular wall and follicular cavity of testes (**D**). NP means nutritive phagocytes.

In the gonads of *S*. *intermedius* fed *S*. *horneri*, the primary oocytes only attached to the follicular wall of ovaries (Fig. 2A) and sperms only occurred in the follicular wall of testes (Fig. 2B). Regarding the gonads of sea urchins fed *S*. *japonica* at the same period, the oocytes were detached from the wall and gradually replaced nutritive phagocytes in the follicular cavity (Fig. 2C). Consistently, the basophilic clusters of sperm with a length of ~ 2 μm occurred in both the follicular wall and follicular cavity of testes (Fig. 2D).

### Experiment II

#### Moderately elevated temperatures

**Figure 3:**
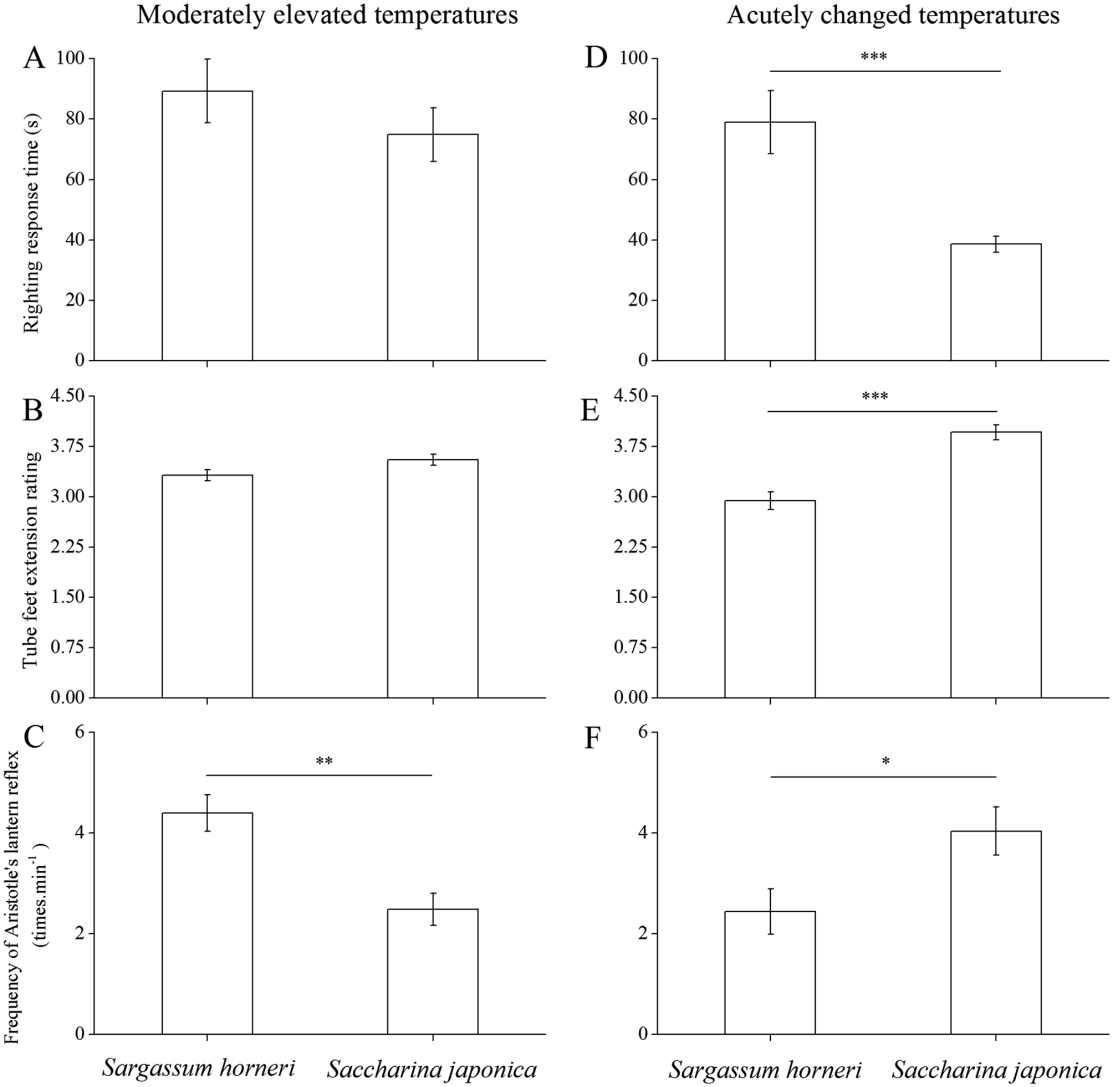
Righting response time (mean ± SD, N = 15; **A**,**D**), tube feet extension rating (mean ± SD, N = 15; **B**,**E**) and Aristotle's lantern reflex (mean ± SD, N = 7 for experimental group and N = 10 for control group at moderately elevated temperatures; (**C**) mean ± SD, N = 10 for both groups; (**F**) in either treatment in respond to moderately elevated or acutely changed water temperatures. *Sargassum horneri* and *Saccharina japonica* refer to the experimental and control groups, respectively. Moderately elevated water temperatures refers to seawater temperature rose from 23.5 to 26.5 °C at a rate of 0.5 °C per day and maintained for 1 week. To simulate the changes of water temperature in Haiyang island near Dalian (39° 03′ N, 123° 09′ E) where water temperature frequently fluctuates from 22 to 16 °C instantly by the cold water mass, sea urchins were transferred quickly from 23.5 to 15 °C, maintaining at 15 °C for an hour and subsequently quickly returned to 23.5 °C for another hour to finish one cycle of acutely changed temperatures. After four cycles, these behaviors were observed. The asterisks *,** and *** mean *P* < 0.05 and *P* < 0.01 mean *P* < 0.001.

Significant difference was not found between the treatments in either righting response time (89.3 ± 57.6 s for *S*. *intermedius* fed *S*. *horneri*, 74.9 ± 48.5 s for sea urchins fed *S*. *japonica*, Kruskal–Wallis *H* = 1.182, *P* = 0.277; Fig. 3A) or tube feet extension rating (3.32 ± 0.93 for *S*. *intermedius* fed *S*. *horneri*, 3.55 ± 0.90 for individuals fed *S*. *japonica*, Kruskal–Wallis *H* = 3.814, *P* = 0.051; Fig. 3B). However, Aristotle's lantern reflex of *S*. *intermedius* fed *S*. *horneri* (4.40 ± 0.95 times min^−1^) was significantly higher than those fed *S*. *japonica* (2.49 ± 1.39 times min^−1^, *t* = 3.343, *P* = 0.003, Fig. 3C).

#### Acute changes in water temperatures

The righting response time of *S*. *intermedius* fed *S*. *horneri* (78.9 ± 49.9 s) was significantly higher than those fed *S*. *japonica* (38.6 ± 11.6 s, Kruskal–Wallis *H* = 17.149, *P* < 0.001, Fig. 3D). Consistently, tube feet extension rating (3.96 ± 1.05) and Aristotle's lantern reflex (4.04 ± 1.87 times min^−1^) of individuals fed *S*. *japonica* were significantly greater than those fed *S*. *horneri* (2.94 ± 1.27, Kruskal–Wallis *H* = 33.872, *P* < 0.001 for tube feet extension rating, Fig. 3E; 2.44 ± 1.94 times min^−1^, Kruskal–Wallis *H* = 6.281, *P* = 0.012 for Aristotle's lantern reflex, Fig. 3F).

## Discussion

The increased incidence of bacteria causes black-mouth and spotting diseases greatly decreased the production of *S*. *intermedius* aquaculture^1,4,8^. We found that *S*. *intermedius* fed *S*. *horneri* showed significantly fewer morbidity than those fed *S*. *japonica*. A reasonable explanation is that polysaccharides enriched in *S*. *horneri*^30^ stimulate the innate immune system of sea urchins because cell walls of pathogenic bacteria possess polysaccharides that are identified as characteristic antigen molecules by the innate immune system^31–33^. This indicates that *S. japonica* could be an effective approach to the disease prevention in *S*. *intermedius*.

*Strongylocentrotus intermedius* necessarily requires at least one summer to develop from fertilized eggs to adults of commercial size (> 5 cm of test diameter) in long line culture in China^4,7^. It is not essential for small sea urchins to develop gametes, but more appropriate for somatic growth in aquaculture^34^. Gonadal precocity greatly consumes the stored energy and consequently leads to poor somatic growth^35,36^ and probable mortality of sea urchins. Dietary protein is the basis of gonadal developments of sea urchins^35,37^. The present study showed that *S*. *intermedius* fed *S*. *horneri* exhibited slower gonadal development than those fed *S*. *japonica*, even though no significant difference of crude protein concentration were found between the two brown algae. This indicates that gonadal development of sea urchins is probably due to other nutrient elements. Collectively, the present study indicates that *S*. *horneri* is an effective food to avoid the precocious puberty of *S*. *intermedius* and may subsequently contribute to their energy storage.

In addition, cultured *S. intermedius* requires higher resistance ability at adverse water temperatures in summer. Behaviors, which are realized by the coordination of neuromuscular systems^38,39^, display a strong correlation with the fitness of sea urchins^40^. Water temperature significantly affects neuromuscular activities. For example, the sea urchin *Strongylocentrotus purpuratus* showed a decreased adhesion when being exposed to the elevated water temperatures^41^. For the first time, the present study found that different species of brown algae have different effects on sea urchin behaviors under acutely changed and moderately elevated temperatures. Specifically, sea urchins fed *S*. *horneri* showed significantly greater Aristotle's lantern reflex than those fed *S*. *japonica* when being exposed to moderately elevated temperatures (an increase from 23.5 to 26.5 °C at a rate of 0.5 °C per day and maintained at 26.5 °C for 1 week). The Aristotle's lantern reflex represents the ability to operate sea urchin jaws to grasp a food around and is commonly used as an indicator for the food intake capacity^29,42^. Consistently, *S*. *intermediu*s fed *S*. *horneri* exhibited significantly higher food consumption than those fed *S*. *japonica*. The study indicates that *S. intermedius* fed *S*. *horneri* displays a significantly greater capacity in thermal tolerance than those fed *S*. *japonica*. In comparison, sea urchins fed *S*. *japonica* outperformed those fed *S*. *horneri* in behaviors of righting, tube feet extension and Aristotle's lantern reflex under acutely changed water temperatures. These findings clearly suggest that *S*. *intermedius* fed *S. japonica* is more suitable for the areas with cold water mass in summer because it probably reduces the negative impacts of acute changes in water temperature on sea urchins. A possible explanation is that *S. japonica* contributes to the reduction of the free-radical level in organisms^43^, decreases tissue hypoxia and subsequently improves the neuromuscular activities of sea urchins.

In conclusion, *S*. *horneri* has direct application potential for the long line culture of *S*. *intermedius* in summer, because of the advantages in health, energy storage and resistance abilities. Further, *S*. *japonica* is appropriate for *S*. *intermedius* aquaculture in the areas with cold water mass where acute changes of water temperature exist. The present study provides valuable information into the management of the long line culture of small *S*. *intermedius* in summer.

## Materials and methods

### Sea urchins

Experimental sea urchins were produced in November 2018, fed *Ulva pertusa *ad libitum for ~ 2 months until the test diameter reached 0.3–0.4 cm diameter and subsequently fed *S*. *japonica* for culture^1,4^. Three hundred healthy *S*. *intermedius* (~ 3 cm of test diameter) were randomly selected from an aquaculture farm in Huangnichuan, Dalian (121° 45′ N, 38° 82′ E) and then were transported to the Key Laboratory of Mariculture and Stock Enhancement in North China’s Sea, Ministry of Agriculture at Dalian Ocean University (121° 56′ N, 38° 87′ E) on 9 July 2019. Sea urchins were maintained in a large fiberglass tank (length × width × height: 180 × 100 × 80 cm) of the recirculating system (Huixin Co., Dalian, China) to acclimatize to laboratory conditions and fed *S*. *japonica *ad libitum for 1 week with aeration. Water quality parameters were measured daily. Water temperature was 23.55 ± 0.07 °C, pH 7.72 ± 0.02 and salinity 33.76 ± 0.04. They were then fasted for another week until the experiment began.

Test diameter, wet body weight and wet gonad weight were evaluated for the initial conditions of sea urchins before the experiments started (N = 20 for test diameter and wet body weight; N = 10 for wet gonad weight).

### Crude protein, fiber, fat and ash of *S*. *horneri* and *S*. *japonica*

Samples were taken from each dried brown alga to investigate their organic composition (crude protein, crude fiber and crude fat) and ash on 20 August 2019 (N = 3). Semi-micro Kjeldahl nitrogen was used to determine the crude protein concentration of the dried brown algae^44^. In order to measure the crude fiber concentration of brown algae, about 10 g of each sample of the dried brown algae was boiled with a mixed solution (1.25% dilute acid and dilute alkali) for 30 min and ashed at 550 °C to remove the minerals^45^. Five grams of each dried sample and 15 mL petroleum ether were added to the Soxhlet extractor and refluxed at constant temperature (45 ± 1 °C) for eight hours to assess the crude fat concentration of the brown algae^46^. To investigate the ash concentration of the brown algae, approximate two g dried samples were placed in a constant weight fritted glass and burned in a muffle furnace (M110, Thermo CO., U.S) at 550 °C for 48 h^45^.

### Experiment I

#### Experimental design

Diet was the experimental factor, either *S*. *horneri* or *S*. *japonica*. Fresh *S*. *horneri* were collected from a farm in Huangnichuan Dalian (121° 45′ N, 38° 82′ E) and *S*. *japonica* from Dalian Bay (120° 37′ E 38° 56′ N) in July 2019. Individuals were fed dried *S*. *horneri* (experimental group) and dried *S*. *japonica* (control group) ad libitum for ~ 9 weeks during the experiment (from 23 July 2019 to 25 September 2019). One large fiberglass tank was used for each experimental treatment. One hundred sea urchins were haphazardly chosen and put into 100 individual cylindrical cages (length × width × height: 10 × 10 × 20 cm; 1.5 cm of mesh size) in each tank (length × width × height: 150 × 100 × 60 cm) of the recirculating system (Huixin Co., Dalian, China) with aeration, according to the experimental design. Diseased sea urchins were removed timely from the tanks to avoid the potential spread of infectious diseases in experimental treatments and were transported into new tanks (length × width × height: 75 × 45 × 35 cm) for individual culture and observation following with the previous management.

Water temperature was not controlled, ranging from 21.3 to 25.6 °C during the experiment. Water quality parameters were measured weekly as pH 7.59–7.85 and salinity 32.69–32.13. One-half of the seawater was renewed daily.

#### Number of survived and diseased sea urchins

**Figure 4:**
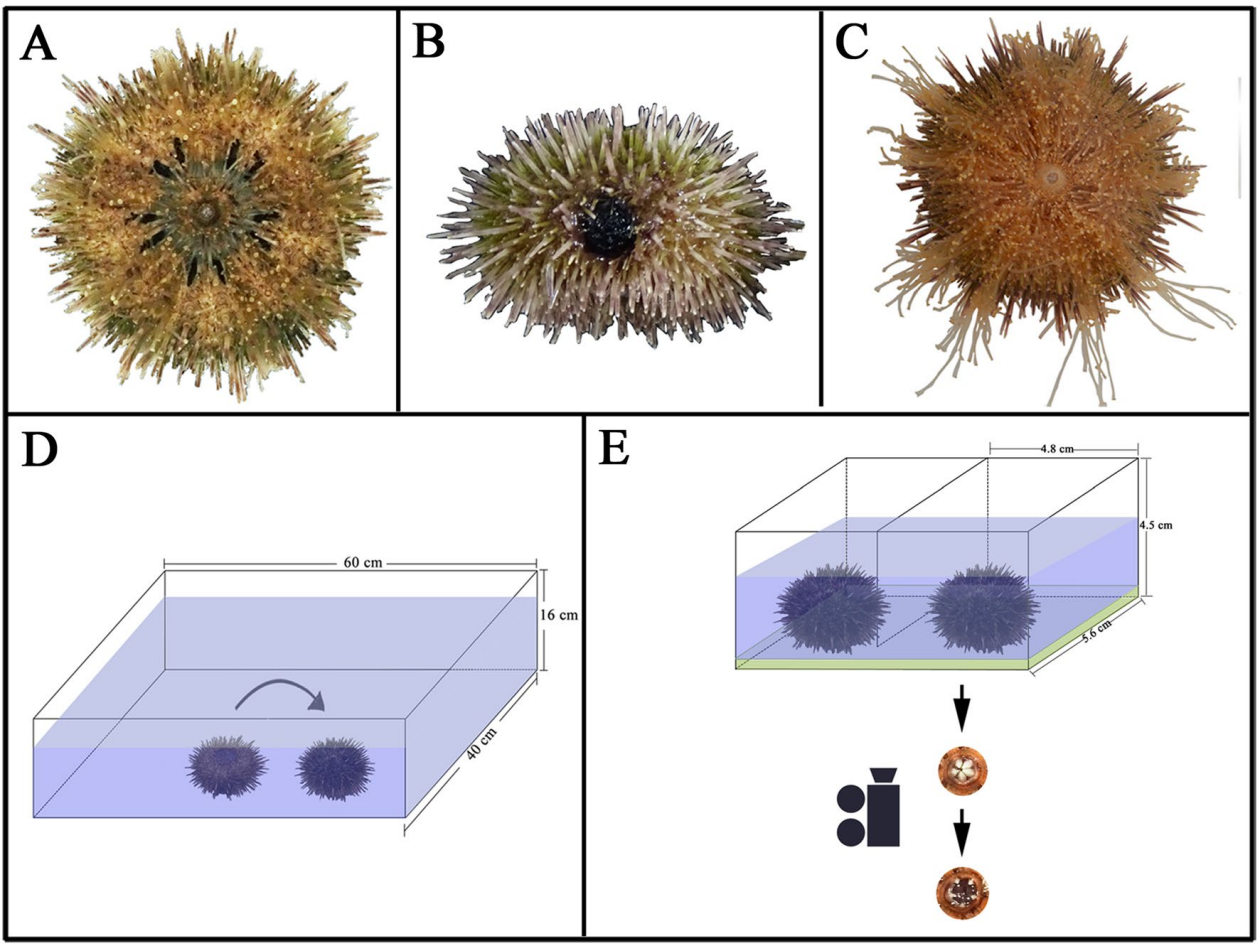
The conceptual diagrams show the black-mouth disease (**A**), spotting disease (**B**) and without disease performance (**C**) of sea urchin as well as the devices for righting behavior (**D**) and Aristotle's lantern reflex (**E**).

Black-mouth disease refers to the perioral membrane turns black (Fig. 4A) with the decreased ability of attaching and feeding in sea urchins^47^. Sea urchin with spotting disease is indicated by the spotting lesions with red, purple or blackish color on the body wall followed by the detachment of local spines^48^ (Fig. 4B). The enlarging spotting lesions commonly cause ulceration on the body wall and finally result in death^8^. Sea urchin without disease performance is shown in Fig. 4C. The number of survived and diseased sea urchins (either black-mouth or spotting diseased) was recorded during the experiment.

#### Dried food consumption

The measurement of food consumption was conducted for six consecutive days (from 7 August 2019 to 12 August 2019). The total supplemented and remained diets were weighed (G & G Co., San Diego, USA) after removing the water on their surface. The samples of uneaten diets were collected, weighed and dried for 4 days at 80 °C and then reweighed (N = 5). To avoid the loss of uneaten food, a fine silk net (mesh size 260 μm) was set outside the cage to collect the fragments of uneaten brown algae^7^.

Dried food consumption was calculated as follows (according to Zhao et al.^49^ with some revisions):

$$\text{F } = {\text{ W}}_{1}\times ({1}-\frac{{\text{B}}_{\text{s}}-{\text{B}}_{\text{u}}}{{\text{B}}_{\text{s}}}\text{)}-{\text{W}}_{2} \times (1-\frac{{\text{C}}_{\text{s}}-{\text{C}}_{\text{u}}}{{\text{C}}_{\text{s}}}\text{)}$$

F = dried food consumption (g), W_1_ = wet weight of total supplement diets (g), W_2_ = wet weight of total uneaten diets (g), B_s_ = wet weight of sample supplemented diets (g), B_u_ = dry weight of sample supplemented diets (g), C_s_ = wet weight of sample uneaten diets (g), C_u_ = dry weight of sample uneaten diets (g).

#### Growth

Test diameters, Aristotle’s lantern length were measured using a digital vernier caliper (Mahr Co., Ruhr, Germany). Body, Aristotle's lantern and gut were weighted wet using an electric balance (G & G Co., San Diego, USA) on 25 September 2019 (N = 27 for test diameter and wet body weight; N = 6 for Aristotle's lantern length, wet weight of Aristotle's lantern and gut).

Specific growth rate (SGR) was calculated according to the following formula:

$${\text{SGR}}\text{ (}{\%}\text{)} \, \text{=}\frac{\ln{\text{P}}_{2}-\ln{\text{P}}_{1}}{\text{D}}\,\times\,{100}$$

SGR = specific growth rate, P_2_ = final wet body weight, P_1_ = initial wet body weight, D = experimental duration.

#### Gonad yield

Gonads were carefully collected from each treatment and weighed using an electric balance (G & G Co., San Diego, USA) on 25 September 2019 (N = 6). Gonad index was calculated according to the following formula:

$$\text{GI } (\%)= \text{ } \frac{\text{GW}}{{\text{BW}}}\,\times\,{100}$$

GI = gonad index, GW = wet gonad weight, BW = wet body weight.

#### Gonadal development

One of five pieces of each gonad was preserved in the Bouin’s solution (saturated picric acid solution: formaldehyde: glacial acetic acid = 15: 5: 1) for 48 h between the treatments (N = 6). Standard histology technique, including embedment, infiltration, section and stain, was performed to make the gonad tissue slices^50^. Sections were classified according to the stage of development of germinal cells and nutritive phagocytes: stage I, recovering; stage II, growing; stage III, premature; stage IV, mature; stage V, partly spawned; stage VI, spent^51–53^.

### Experiment II

#### Experimental design

Experiment II lasted for 4 weeks (from 25 September 2019 to 23 October 2019). Eighty healthy sea urchins were haphazardly selected from each treatment at the end of experiment I. They were then distributed into 80 cylindrical cages (5 × 10 × 10 cm) in each fiberglass tanks (length × width × height: 77.5 × 47.0 × 37.5 cm) of the temperature-controlled system (Huixin Co., Dalian, China) with aeration in both treatments. Sea urchins were maintained at 23.5 °C for 2 weeks (the average water temperature of experiment I) to eliminate the past thermal history, following the previous diet strategy of experiment I. Water quality was recorded daily as pH 7.83–7.85 and salinity 32.44–32.62. One-third of the seawater was renewed daily.

**Figure 5:**
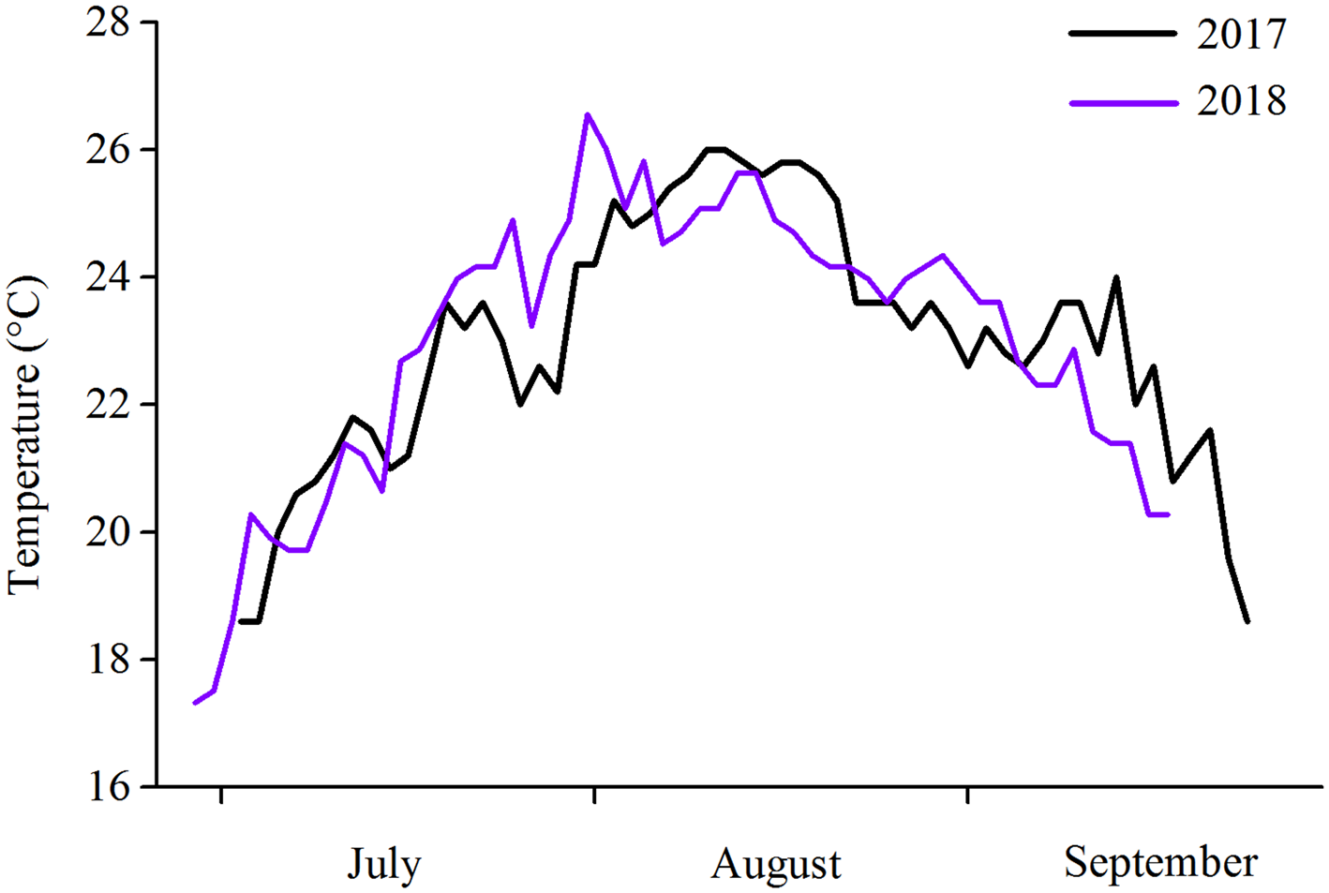
Daily records of the water temperature in Heishijiao sea area, Dalian (~ 2 m water depth, 38° 51′ N, 121° 33′ E) in the summers of 2017 and 2018.

Subsequently, to investigate whether *S. horneri* and *S. japonica* contribute to the resistance abilities of small *S. intermedius* at moderately elevated temperatures, 40 individuals were haphazardly chosen from each treatment and placed into 40 cylindrical cages (5 × 10 × 10 cm) in each tank (length × width × height: 77.5 × 47.0 × 37.5 cm) of a temperature-controlled system (Huixin Co., Dalian, China) with aeration for both groups on 9 October 2019. They were subsequently exposed to the moderately elevated temperatures (rose from 23.5 to 26.5 °C at a rate of 0.5 °C per day and maintained at 26.5 °C for 1 week), according to the records of water temperature in Heishijiao sea area (~ 2 m water depth, 38° 51′ N, 121° 33′ E) in the summer of 2017 and 2018 (Fig. 5). Righting behavior, tube feet extension and Aristotle's lantern reflex were assessed on 23 October 2019.

Similarly, to explore the effects of *S*. *horneri* and *S*. *japonica* on the resistance abilities of small *S*. *intermedius* under acute changes of water temperature, another 40 individuals were randomly selected and placed into 40 cylindrical cages (5 × 10 × 10 cm) in each tank (length × width × height: 77.5 × 47.0 × 37.5 cm) of the temperature-controlled system (Huixin Co., Dalian, China) with aeration for both treatments on 9 October 2019. The water temperature was set at 23.5 °C. A tank of seawater was prepared at 15 °C. To simulate the changes of water temperature in Haiyang island near Dalian (39° 03′ N, 123° 09′ E) where water temperature frequently fluctuates from 22 to 16 °C instantly by the cold water mass^17^, sea urchins were transferred directly from 23.5 to 15 °C, maintained at 15 °C for an hour and subsequently quickly returned to 23.5 °C for another hour to finish one cycle of the acute change of water temperature. After four cycles, righting behavior, tube feet extension and Aristotle's lantern reflex of sea urchins were observed.

#### Righting behavior

Sea urchins were placed with the aboral side down on the bottom of an experimental tank (length × width × height: 60 × 40 × 16 cm, Fig. 4D). Righting response time is the time required for individuals in the inverted posture to right themselves with the aboral side up^22^. The righting response time in seconds was recorded during 10 min. If individuals did not right themselves within 10 min, the time was counted as 600 s (N = 15).

#### Tube feet extension

The method of assessing tube feet extension was established according to You et al.^27^, with some revisions. Sea urchins were maintained in a tank (length × width × height: 12 × 10 × 10 cm) with fresh seawater for ~ 5 min before the observation (N = 15). The subjective assessment of tube feet extension was evaluated by a well-trained team (5 persons) that was familiar with tube feet extension analysis of sea urchins. The ranking method was quantified based on the quantity and length of tube foot.

Tube feet extension (rating 1–5):

- 1 = extremely abnormal (not extending)
- 2 = severe abnormality (extremely low quantity and extremely short length)
- 3 = moderate anomaly (low quantity and short length)
- 4 = mild abnormality (slight decrease in quantity and length)
- 5 = normal (normal quantity and length)

#### Aristotle's lantern reflex

A simple device, which has two small compartments (length × width × height: 4.8 × 5.6 × 4.5 cm) with a food film on the bottom, was used to measure Aristotle's lantern reflex according to our previous study^29^. Food film was made by a mixture of ~ 2.5 g agar and 50 ml seawater in order to avoid the potential impacts of the food palatability on sea urchins. The number of Aristotle's lantern reflex were counted within 5 min using a digital camera (Canon Co., Shenzhen, China) under the device (N = 7 for sea urchins fed *S*. *horneri* and N = 10 for individuals fed *S*. *japonica* under moderately elevated temperatures; N = 10 for both groups under acutely changed temperatures; Fig. 4E).

### Statistical analysis

Normal distribution and homogeneity of variance of the data were analyzed using the Kolmogorov–Smirnov test and Levene test, respectively. The number of survived and diseased *S*. *intermedius* were compared using the Fisher′s exact test. Food consumption was analyzed using one-way repeated measured ANOVA. Kruskal–Wallis test was performed to compare the difference of righting behavior and tube feet extension between the treatments and also used to analyze Aristotle's lantern reflex under acutely changed temperatures. Independent-samples t test was carried out to compare the difference between the final and initial conditions of sea urchins. Test diameter, wet body weight, SGR, Aristotle's lantern length, wet Aristotle's lantern weight, wet gut weight, gonad index and Aristotle's lantern reflex (under moderately elevated temperatures) were analyzed using the independent-samples t test. All data analyses were performed using SPSS 19.0 statistical software. A probability level of *P* < 0.05 was considered significant.
